# *Bacillus cytotoxicus* Genomics: Chromosomal Diversity and Plasmidome Versatility

**DOI:** 10.3389/fmicb.2021.789929

**Published:** 2021-12-09

**Authors:** Nancy Fayad, Klèma Marcel Koné, Annika Gillis, Jacques Mahillon

**Affiliations:** ^1^Laboratory of Food and Environmental Microbiology, Earth and Life Institute, Louvain-la-Neuve, Belgium; ^2^School of Pharmacy, Lebanese American University, Byblos, Lebanon

**Keywords:** *Bacillus cereus*, *Bacillus cytotoxicus*, conjugation, mobile genetic elements, plasmid

## Abstract

*Bacillus cytotoxicus* is the thermotolerant representative of the *Bacillus cereus* group. This group, also known as *B. cereus sensu lato*, comprises both beneficial and pathogenic members and includes psychrotolerant and thermotolerant species. *Bacillus cytotoxicus* was originally recovered from a fatal outbreak in France in 1998. This species forms a remote cluster from the *B. cereus* group members and reliably contains the *cytk-1* gene, coding for a cytotoxic variant of cytotoxin K. Although this species was originally thought to be homogenous, intra-species diversity has been recently described with four clades, six random amplified polymorphic DNA (RAPD) patterns, and 11 plasmids profiles. This study aimed to get new insights into the genomic diversity of *B. cytotoxicus* and to decipher the underlying chromosomal and plasmidial variations among six representative isolates through whole genome sequencing (WGS). Among the six sequenced strains, four fitted the previously described genomic clades A and D, while the remaining two constituted new distinct branches. As for the plasmid content of these strains, three large plasmids were putatively conjugative and three small ones potentially mobilizable, harboring coding genes for putative leaderless bacteriocins. Mobile genetic elements, such as prophages, Insertion Sequences (IS), and *Bacillus cereus* repeats (*bcr*) greatly contributed to the *B. cytotoxicus* diversity. As for IS elements and *bcr*, IS*3* and *bcr1* were the most abundant elements and, along with the group II intron *B.c.I8*, were found in all analyzed *B*. *cytotoxicus* strains. When compared to other *B. cytotoxicus* strains, the type-strain NVH 391-98 displayed a relatively low number of IS. Our results shed new light on the contribution of mobile genetic elements to the genome plasticity of *B. cytotoxicus* and their potential role in horizontal gene transfer.

## Introduction

*Bacillus cereus* group, also named *B. cereus sensu lato (s.l.)*, contains closely related Gram-positive, facultative aerobe, and endospore-forming bacteria. It includes *B. cereus sensu stricto* (*s.s*.), *Bacillus thuringiensis*, *Bacillus anthracis*, *Bacillus mycoides*, *Bacillus pseudomycoides*, *Bacillus weihenstephanensis*, and *Bacillus cytotoxicus*. Recently though, more species have been proposed as new members, such as *Bacillus toyonensis* (Jimenez et al., 2013), *Bacillus wiedmannii* (Miller et al., 2016), *Bacillus gaemokensis* (Jung et al., 2010), *Bacillus bingmayongensis* (Liu et al., 2014), or *Bacillus manliponensis* (Jung et al., 2011). The group gathers member species from various ecological niches that display a large spectrum of virulence, from the insect pathogen *B. thuringiensis*, used worldwide as biopesticide and plant protection agent, to human pathogenic strains of *B. anthracis* or emetic *B. cereus* (Agata et al., 1995; Turnbull, 1999). It also spans from psychrotrophic (growth at temperature below 7°C) to thermotolerant (growth up to 53°C) strains (Stenfors Arnesen et al., 2008).

Some strains of *B. cereus s.l*. have been implicated in food outbreaks associated with emetic or diarrheic syndromes. The former is due to cereulide, a thermostable, protease and pH-resistant dodecadepsipeptide toxin preformed in food, whose genetic determinants are plasmid-borne (Agata et al., 1994; Ehling-Schulz et al., 2004; Hoton et al., 2005). The diarrheal syndrome is presumably caused by one or more potential enterotoxins. In fact, *B. cereus* can produce several potential enterotoxins, including hemolysin BL (HBL), non-hemolytic enterotoxin (Nhe), enterotoxin FM (EntFM), and cytotoxin K (CytK) (Granum, 1994; Lund et al., 2000; Senesi and Ghelardi, 2010). The latter was originally discovered in a *B. cereus* strain (NVH 391-98) isolated from an outbreak that caused the death to three elderly persons in France in 1998 (Lund et al., 2000). This strain was further shown to be able to grow up to 53°C. The name *B. cytotoxicus* was coined for this new thermotolerant “*B. cereus*” strain (Guinebretière et al., 2013). Due to their ability to thrive at relatively high temperatures, strains of *B. cytotoxicus* have recently been isolated in geothermal waters (Cavello et al., 2020). Some *B. cytotoxicus* isolates, including strain NVH 391-98, were shown to over-produce the CytK toxin, as compared to other strains of the same species (Heini et al., 2018). Two variants of cytotoxin K have now been described: CytK-1 and CytK-2. The former is associated with *B. cytotoxicus* and is more cytotoxic than the second, which is found in some mesophilic strains of *B. cereus* (Fagerlund et al., 2004).

*Bacillus cereus* members have been classified into seven phylogenetic groups according to *panC* typing. All emetic *B. cereus* and thermotolerant *B. cytotoxicus* strains fall in groups III and VII, respectively (Guinebretière et al., 2008). These seven groups can also be organized in three genomic clades based on their core and pan-genomes. Interestingly though, the *B. cytotoxicus* strains form a distinct cluster among the *B. cereus* members (Fagerlund et al., 2007; Auger et al., 2008; Guinebretière et al., 2008; Bazinet, 2017). This *B. cytotoxicus* diversity was further supported in recent studies in which isolated strains were classified into four genomic clades (A–D; Stevens et al., 2019). Also, a set of strains isolated from food products were classified into six random amplified polymorphic DNA (RAPD) groups and 11 plasmid profiles (Koné et al., 2019). Although the diversity within the *B. cytotoxicus* species is now well established, the extent of these genetic and genomic variations remains poorly characterized. The aim of this study was to further explore the genomic and genetic diversity of six *B. cytotoxicus* strains pertaining to different genetic profiles. Whole genome sequences of these strains were compared to those of other *B. cytotoxicus* genomes publicly available. Sequences of chromosomal indels, mobile elements, and extrachromosomal molecules were also analyzed in detail.

## Materials and Methods

### Strains and Genomes

The strains used in this study for whole genome sequencing (WGS) originated from either potato flakes (E8.1, E17.4, E28.3, and PDT2.12) or instant soup (SM1.1 and SM2.8). They were selected according to their RAPD pattern, plasmid profile, and origin, as previously described (Koné et al., 2019). The genome sequences of strains NVH 391-98 (BioProject: PRJNA13624; Lapidus et al., 2008), CH_1 and CH_2 (BioProject: PRJNA394959; Stevens et al., 2019), and AFSSA_08CEB44bac (BioProject: PRJEB14962) were used as representatives of clades A, B, C, and D, respectively. The nucleotides sequences of these strains were retrieved from NCBI genome Refseq database.^1^ Although more genomes are publicly available, only one representative of each clade was retrieved for the analysis. The relevant features of the *B. cytotoxicus* strains used in this study are shown in Table 1.

**Table 1:** Origin and main features of the *Bacillus cytotoxicus* strains used in this study.

Strains	RAPD/plasmid profiles^a^	Origin	Reference
E8.1	A/PP10	Potato flake	Koné et al., 2019
E17.4	E/PP2	Potato flake	
E28.3	A/PP8	Potato flake	
PDT2.12	F/PP4	Potato flake	
SM1.1	D/PP9	Instant soup	
SM2.8	D/PP2	Instant soup	
**Genomes of representative strains retrieved from GenBank**			
**Strains**	**Genomic clade^b^**	**Origin**	**Reference**
NVH 391–98	A	Vegetable soup	Lapidus et al., 2008; Guinebretière et al., 2013
CH_1	B	Potato flake	Stevens et al., 2019
CH_2	C	Potato flake	
AFSSA_08CEB44Bac44	D	Semolina	BioProject: PRJEB14962

According to Koné et al. (2019)^a^ and Stevens et al. (2019).^b^

### DNA Extraction and WGS

A single fresh colony from a LB agar plate incubated at 30°C was cultured overnight in liquid LB medium (120rpm at 30°C). Genomic DNA extraction was performed using the Wizard Genomic DNA purification kit (Promega, United States). The quality of the DNA extraction was checked with the spectrophotometer Nanodrop 1000 (ThermoFisher Scientific, Wilmington, DE, United States) and on 0.8% agarose gel electrophoresis.

The complete genomes of isolates SM2.8 and E28.3 were first sequenced by Illumina Miseq (Illumina, San Diego, CA, United States), with paired-end run (2×300pb), followed by MinION technology sequencing (Oxford Nanopore, United Kingdom). For Illumina sequencing, a *de novo* assembly was conducted with SPAdes assembler software v3.10.1 (Bankevich et al., 2012) followed by a mapping with BWA-MEM version 0.7.12-r1039.^2^ SPAdes 3.13.0 software was then used to combine the Illumina and MinION data (Antipov et al., 2015). The demultiplexing and adapter sequence trimming were performed with Porechop v0.2.4.^3^ Isolates E8.1, E17.4, PDT2.12, and SM1.1 were first sequenced using PacBio (Pacific Biosciences, CA, United States) and polished with Miseq technology sequencing. Reads were *de novo* assembled with Fly 2.6 software (Kolmogorov et al., 2019). For polishing, paired-end sequences were trimmed with BBDuk^4^ and assembled with SPAdes-3.13.0 (Antipov et al., 2015). Newly sequenced genomes completeness was assessed using Benchmarking Universal Single-Copy Orthologs (BUSCO; Seppey et al., 2019).

On average per genome, read depth ranged between 64 and 187, with read lengths N50/N90 between 9718/7246 and 16537/8431. According to BUSCO v5.0 genome completeness assessment, E17.4 and E8.1 showed coverage scores of 98.68 and 99.76%, respectively, while the remaining four strains showed coverage scores of 100%. Genomes were then annotated using Rapid Annotations using Subsystems Technology (RAST) web-based tool (Aziz et al., 2008). The genome sequences and annotation for the six *B. cytotoxicus* strains sequenced in this work have been deposited at NCBI under BioProject number PRJNA684687.

### Bacterial Conjugation

As potentially conjugative plasmids were found in the *B. cytotoxicus* E8.1 and E28.3 strains (see below), a filter-mating conjugation was performed to assess their self-transferability. *Bacillus cytotoxicus* strains carrying the putative conjugative plasmids were used as donors (E8.1 for plasmids pE81-84 and pE81-53, and E28.3 for pE283-80), while strain E17.4, which did not carry those plasmids, was used as recipient. Spontaneous streptomycin-resistant mutants of the donor (100μgml^−1^) and rifampicin-resistant mutants (50μgml^−1^) of recipient strains were used in filter-mating experiments as previously described by Hinnekens et al. (2019). After the mating, 100 CFUs of the potential transconjugants were PCR-screened for the presence of the conjugative plasmids.

### Bioinformatic Analysis for Chromosomal and Plasmidial Diversity

Mauve alignment software (Darling et al., 2010) was used to align newly sequenced genomes against representatives of the four clades previously described (Stevens et al., 2019). *Bacillus cytotoxicus* strain AFSSA_08CEB44bac was excluded from the subsequent analysis due to its incomplete genome assembly. To assess their relatedness, single nucleotide polymorphisms (SNPs) were also extracted as previously described using the web-based tools CSI Phylogeny v1.4 (Kaas et al., 2014). Using MEGA X software (Kumar et al., 2018), the SNPs output files were used to establish a SNP-based phylo-dendrogram with 500 bootstrap replicates.

The average nucleotide identity (ANI), a measure of nucleotide-level genomic similarity between the coding regions of two genomes, was evaluated *via* ANIb (ANI evaluation based on BLAST+). A total aligned nucleotides analysis was also done, using the JSpeciesWS Online Service (http://jspecies.ribohost.com/jspeciesws/#home – Ribocon GmbH – Version: 3.7.9; last accessed: November 15, 2021; Richter et al., 2016).

Next, Blast Ring Image Generator (BRIG) software (Alikhan et al., 2011) was used to highlight the Insertions–Deletions (indels) among tested *B. cytotoxicus* genomes. The most different isolates, strains SM1.1 and SM2.8, as indicated by the SNP-based phylo-dendrogram (see below), were used as references. To furthermore explore the indels shown by BRIG, indels or novel regions sequences were retrieved using the web-based tool Panseq (Laing et al., 2010). These sequences were annotated with RAST web-based tool (Aziz et al., 2008). Function-based comparison functionality of RAST was also used to retrieve other functional differences between *B. cytotoxicus* genomes and to find out specific features, or new metabolic pathways. Plasmid sequence comparison was generated using BLAST+ executables (Camacho et al., 2009) and Easyfig software (Sullivan et al., 2011).

Prophages and transposable elements were also analyzed. PHAge Search Tool Enhanced Release (PHASTER) was used to find prophage sequences in chromosome and plasmid sequences (Arndt et al., 2016). In a given DNA region, the encoded phage-related proteins were annotated and the DNA region was hence designated as “questionable,” “partial,” or “intact” according to the number of phage-related proteins and the prophage with the highest number of similar proteins. As for Insertion Sequences (IS), the online tool ISsaga (Insertion Sequence semi-automatic genome annotation; Varani et al., 2011) was used to extract the copy number of complete IS elements followed by a manual verification of the results and the calculation of their percentage in each genome.

*Bacillus cereus* repeats (*bcr*) are 200–400bp DNA fragments with complex secondary structures that are mostly chromosomic and specific to *B. cereus* group. Analysis of *bcr* was done using nucleotide BLAST searches of the consensus *bcr1*–*bcr18* (Kristoffersen et al., 2011) sequences against complete *B. cytotoxicus* genomes. Based on former studies (Tourasse et al., 2006; Kristoffersen et al., 2011) and our observations, the algorithm parameters for MegaBLAST (https://blast.ncbi.nlm.nih.gov/Blast.cgi; v2.8.1; Morgulis et al., 2008) were set as follows: The word size – length of a seed that allows the BLAST engine to initiate an alignment – was set at 16; the opening and extension of a gap were both set at 2; and expect range was set between 0 and 0.1. Hits that covered at least 50% of the sequence length and had a minimum of 75% identity were considered as repeats. The same nucleotide MegaBLAST parameters were used to retrieve known group II introns from the intron database (Candales et al., 2011; http://webapps2.ucalgary.ca/~groupii/; Sept 2020) and *B.th*.I3 (Tourasse and Kolstø, 2008) which was absent from the mentioned database. This includes 27 elements with ORFs coding the Intron Encoded Protein (IEP) and three ORF-less elements.

Finally, potential bioactive compounds clusters were predicted with the online tool antiSmash v5.0 (https://antismash.secondarymetabolites.org/#!/start; Blin et al., 2019).

## Results

In a previous study, Koné et al. (2019) classified a collection of 57 *B. cytotoxicus* strains, isolated from different food products, into six RAPD patterns and 11 plasmid profiles. The six isolates used in the present study were selected on the basis of both their distinct RAPD and/or plasmid profiles. Four strains (E8.1, E17.4, E28.3, and PDT2.12) originated from potato flakes, and two (SM1.1 and SM2.8) were isolated from instant soup. As shown in Table 1, isolates sharing the same RAPD patterns (D for SM1.1 and SM2.8 and A for E8.1 and E28.3) showed different plasmid profiles. As for their plasmid profiles, PDT2.12 had a unique one, while E17.4 shared the same pattern with SM2.8 (Table 1).

As shown in Table 2, the chromosome sizes of the six strains ranged from 4,049,327bp (E17.4) to 4,244,837bp (SM2.8), with GC contents of 35.9–36.0%. As expected from our previous work, all six sequenced isolates contained both small (from none to three) and/or large (from none to two) plasmids with sizes varying from 3,421bp (pE283-3) to 83,570bp (pE81-84). They were all circular, with the exception of pE283-14.

**Table 2:** Genomic features of the six sequenced *B. cytotoxicus* strains.

Strain (Clade)	Chromosome size (bp)	Plasmid	Plasmid size (bp)	Plasmid relevant features
E8.1 (C)	4,132,005	pE81-84	83,570	102 CDS (54 hypothetical proteins) Potentially conjugative Contains a Tn*7*-like element Closely related to pE283-80 Partially related to pPDT212-44 and the 67-kb plasmid (Figure 4) Distantly related to pAW63 (*Bacillus thuringiensis*) and pXO2 (*Bacillus anthracis*)
		pE81-53	53,121	63 CDS (33 hypothetical proteins) Related to pCE3 from *B. paranthracis* Partially related to 53-kb plasmid and pBCM1301 of *B. cereus* (Figure 5) Distantly related to the *Clostridium perfringens* pCW3 conjugation system Conjugative: *ca*. 10^−2^ transconjugant per recipient cells (see experimental data)
E17.4	4,049,237	pE174-12	11,673	26 CDS (22 hypothetical proteins) *Bacillus thuringiensis* pGI3-like replicon Partially related to pSM11-12b and pBC9801 (Figure 3) Fibronectin type III domain-containing protein
E28.3 (C)	4,198,865	pE283-80	79,734	99 CDS (43 hypothetical proteins) Potentially conjugative Contains a Tn*7*-like element Closely related to pE81-84 Partially related to pPDT212-44 and the 67-kb plasmid (Figure 4) Distantly related to pAW63 and pXO2
		pE283-14	14,402	Linear plasmid; putative plasmidial tectivirus
		pE283-4	3,662	10 CDS (nine hypothetical proteins) *Bacillus thuringiensis* sv. *israelensis* pTX14-1-like replicon No *mob*-like gene
		pE283-3	3,421	Four 4 CDS (two hypothetical proteins) Staphylococcal pE194- and pSN2-like replicon Streptococcal pMV158-like and *B. thuringiensis* sv. *israelensis* pTX14-1-like Mob
PDT2.12	4,236,127	pPDT212-44	44,141	56 CDS (27 hypothetical proteins) Contains a Tn*7*-like element Partially related to pE81-84, pE283-80 and the 67-kb plasmid (Figure 4) Distantly related to pAW63 and pXO2
SM1.1 (D)	4,205,722	pSM11-51	51,478	73 CDS (54 hypothetical proteins) Putative plasmidial prophage related to the *Brevibacillus* Jenst and *B. thuringiensis* Phi4J1 prophages
		pSM11-43	43,118	70 CDS (20 hypothetical proteins) Putative plasmidial prophage related to *Listeria* prophage BO25
		pSM11-12a	11,640	16 CDSs (five hypothetical proteins) Identical to pSM28-12a Rep protein of an unknown family Mob protein of the *Streptococcus pneumoniae* pMV158 family Contains the *gakA*, *gakB*, and *gakC* genes of leaderless bacteriocins
		pSM11-12b	11,581	26 CDSs (22 hypothetical proteins, many small ones) Identical to pSM28-12b and partly related to pE174-12 and pBC9801 (Figure 3) Rep protein of an unknown family Fibronectin type III domain protein
SM2.8 (D)	4,244,837	pSM28-12a	11,640	Identical to pSM11-12a
		pSM28-12b	11,581	Identical to pSM11-12b

### Chromosomal Diversity

**Figure 1:**
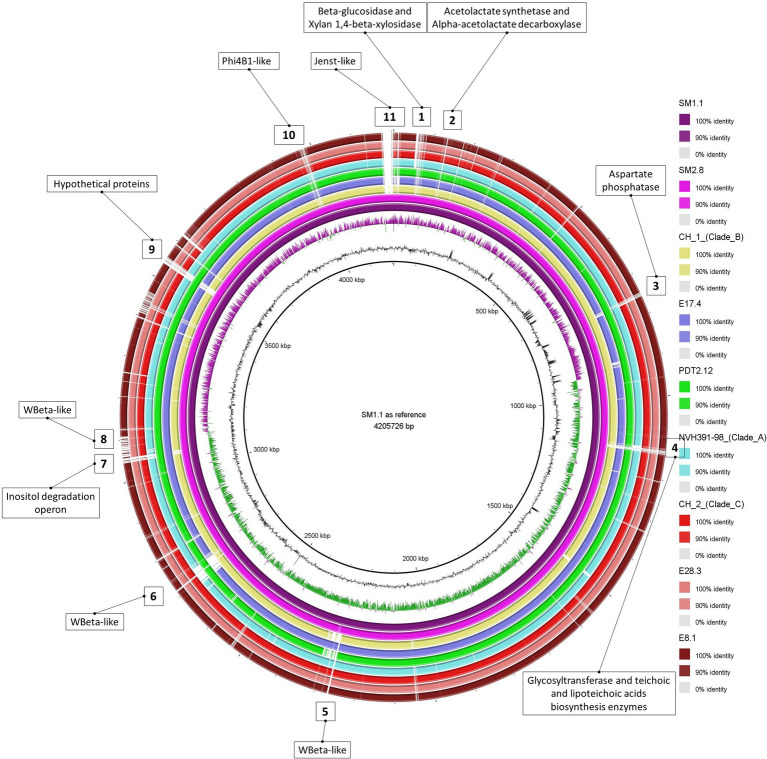
Comparison of nine *B. cytotoxicus* chromosomal sequences. From the center to the periphery: SM1-1 (used as reference), SM2-8, CH_1 (clade B), E17.4, PDT12.12, NVH 391-98 (Clade A), CH_2 (Clade C), E28.3, and E8.1. The white trips represent the indels found in the reference, strain SM1.1, but absent in corresponding strains. Eleven relevant indels are highlighted and annotated.

The chromosomal sequences of the six strains were compared among themselves and with those of NVH 391-98, CH_1, CH_2, and AFSSA_08CEB44Bac44, representatives of clades A–D (Table 1), respectively (Figure 1). SNPs were extracted, with numbers ranging between a minimum of 81 (E8.1 vs. CH_2) and 11,394 (SM1.1 vs. CH_2). A noteworthy remark is that the extracted SNPs were dispersed on the chromosome, not clustered together.

**Figure 2:**
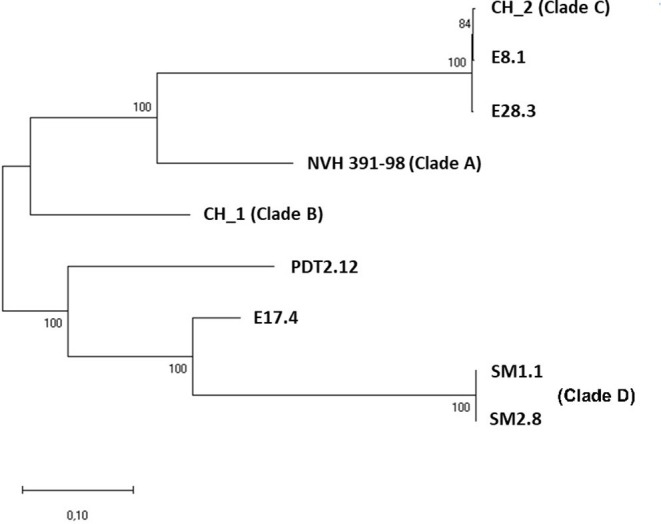
Maximum Likelihood single nucleotide polymorphism (SNP) base-phylogenetic tree of the six sequenced *B. cytotoxicus* strains, along with representatives of genomic clades A (NVH 391-98), B (CH_1), and C (CH_2). Note that clade D also includes strain AFSSA_08CEB44bac. Percentage of corresponding bootstrap replicates shown at the base of each node.

Based on the number of SNPs, a phylo-dendrogram of these strains was also established (Figure 2). The SNP-based phylo-dendrogram showed that strains E8.1 and E28.3 clustered with clade C representatives, while E17.4 and PDT2.12 did not match the previously described clades. The remaining strains, namely the “instant soup” isolates SM1.1 and SM2.8, formed a remote cluster. Using the ANI on the chromosomal level, all pairs of genomes showed ANI values above 99%, except the instant soup strains which had ANI values above 99% with each other, but of *ca*. 98% with the rest of the strains. This is reflected in the phylo-dendrogram: SM1.1 and SM2.8 clustered together, but were phylogenetically distant from the rest.

Using Panseq (Laing et al., 2010), sequence variations among the different strains were retrieved using SM1.1 as reference. As shown in Figure 1, several regions displayed size variations (indicated by squared numbers). They not only included prophages (see below), but also several interesting metabolic loci, such as xylan degradation (#1), acetoin metabolism (#2), (lipo-)teichoic acid synthesis (#4), or inositol degradation (#7) absent from several strains. In addition, function-based comparison of the online tool RAST revealed that strain PDT2.12 harbors genes coding for lactose and galactose uptake and utilization (data not shown).

The analyzed genomes were also mined for potential clusters of bioactive compounds using the online tool antiSmash. Although eight clusters were predicted on the chromosomes of the six *B. cytotoxicus* strains alongside the reference ones NVH 391-98, CH_1, and CH_2, most had no similarity with known clusters, and hence, their existence is questionable. Nevertheless, clusters for the production of fengycin, an antimicrobial lipopeptide (Sur et al., 2018), and bacillibactin, a non-ribosomal peptide (Caulier et al., 2019), were found in all nine strains at similarities of 40 and 46%, respectively. Another cluster found in all analyzed strains is that of the production of heme D1, a molecule produced *via* a non-ribosomal peptide synthetase/Type I PKS polyketide synthase pathway. However, the similarity with a known heme D1 cluster was only at 17%.

### Extrachromosomal Diversity

As for other members of the *B. cereus* group (Gillis et al., 2018), the strains of *B. cytotoxicus* analyzed in this study contain a noticeable number of extrachromosomal elements. As reported in Table 2, all the strains contain from 1 (E17.4 and PDT2.12) to 4 plasmids (E28.3 and SM1.1), with sizes varying from 3,421 to 83,570bp. A list of the relevant features of these *B. cytotoxicus* plasmids can be found in Supplementary Table S1.

#### The Small Plasmids: 3, 4, 12, and 14kb

Strain E28.3 contains two small Rolling-Circle Replicating (RCR) plasmids: pE283-3 (3,421bp) has a replication (*rep*) gene distantly related to those of the staphylococcal plasmids pE194 and pSN2 (Lampson and Parisi, 1986), while pE283-4 (3,662bp) contains a replicon related to that of pTX14-1 from *B. thuringiensis* sv. *israelensis* (Boe et al., 1991). A potential mobilization gene is also present in pE283-3 and shares similarities with the *mob* genes of the streptococcal pMV158 and *B. thuringiensis* sv. *israelensis* pTX14-1 plasmids (Boe et al., 1991). A third small plasmid, pE283-14, is a 14,402bp linear molecule which is likely the prophage state of a tectivirus, as shown for related plasmidial elements found in *B. thuringiensis* (Gillis and Mahillon, 2014b).

Two distinct *ca*. 12-kb plasmids are found in the two closely related strains SM1.1 and SM2.8. Plasmids pSM11-12a (11,640bp) and pSM11-12b (11,581bp) from the former strain are in fact identical to the pSM28-12a and pSM28-12b elements of the latter strain (Table 2). The most striking features of pSM11-12a/pSM28-12a are the presence of a mobilization (*mob*) gene related to that of the streptococcal pMV158 plasmid and a set of three genes coding for putative leaderless, broad spectrum bacteriocins recently described in *Lactococcus garvieae* and other Gram-positive bacteria, including strains of *B. cereus* (Ovchinnikov et al., 2016).

**Figure 3:**
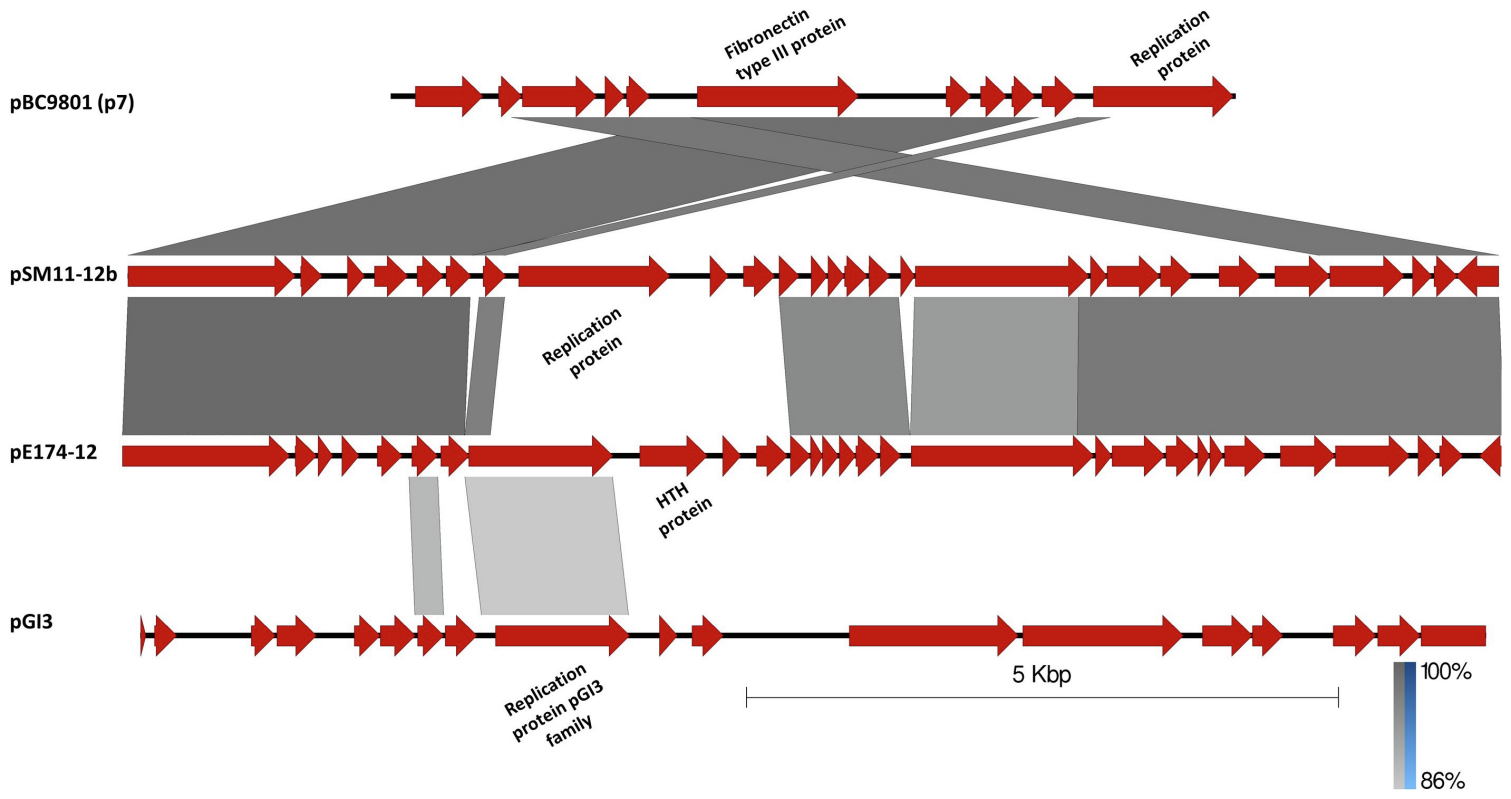
Linear alignment of pBC9801 (also referred to as the 7-kb plasmid in Stevens et al., 2019), pSM11-12b, pE174-12 from *B. cytotoxicus*, and pGI3 from *B. thuringiensis* strain H1.1 (Hoflack et al., 1997; NC_010937.1). CDSs are represented by block arrows. Relevant functions are annotated above or below the corresponding gene. Darkening gray shading reflects increasing nucleotide BLAST sequence identity. Scale and identity percentage are indicated in the lower right-hand corner.

As shown in Figure 3, the pSM11-12b/pSM28-12b plasmid is related to the third 12-kb element, pE174-12 (11,673bp), the only extrachromosomal element of strain E17.4. However, the latter displayed a replication region unrelated to the former but homologous to the replication region of pGI3, another RCR plasmid originating from *B. thuringiensis* strain H1.1 (Hoflack et al., 1997). No other striking features could be found in pE174-12, except the presence of a gene coding for a putative 454-residue fibronectin type III domain containing protein (Table 2). These plasmids are also partially related to pBC9801 (*aka* p7), the 7-kb plasmid from NVH 391-98 (NC_009673.1), the reference type-strain of *B. cytotoxicus* (Figure 3).

#### The Large Plasmids: 43, 44, 51, 53, 80, and 84kb

**Figure 4:**
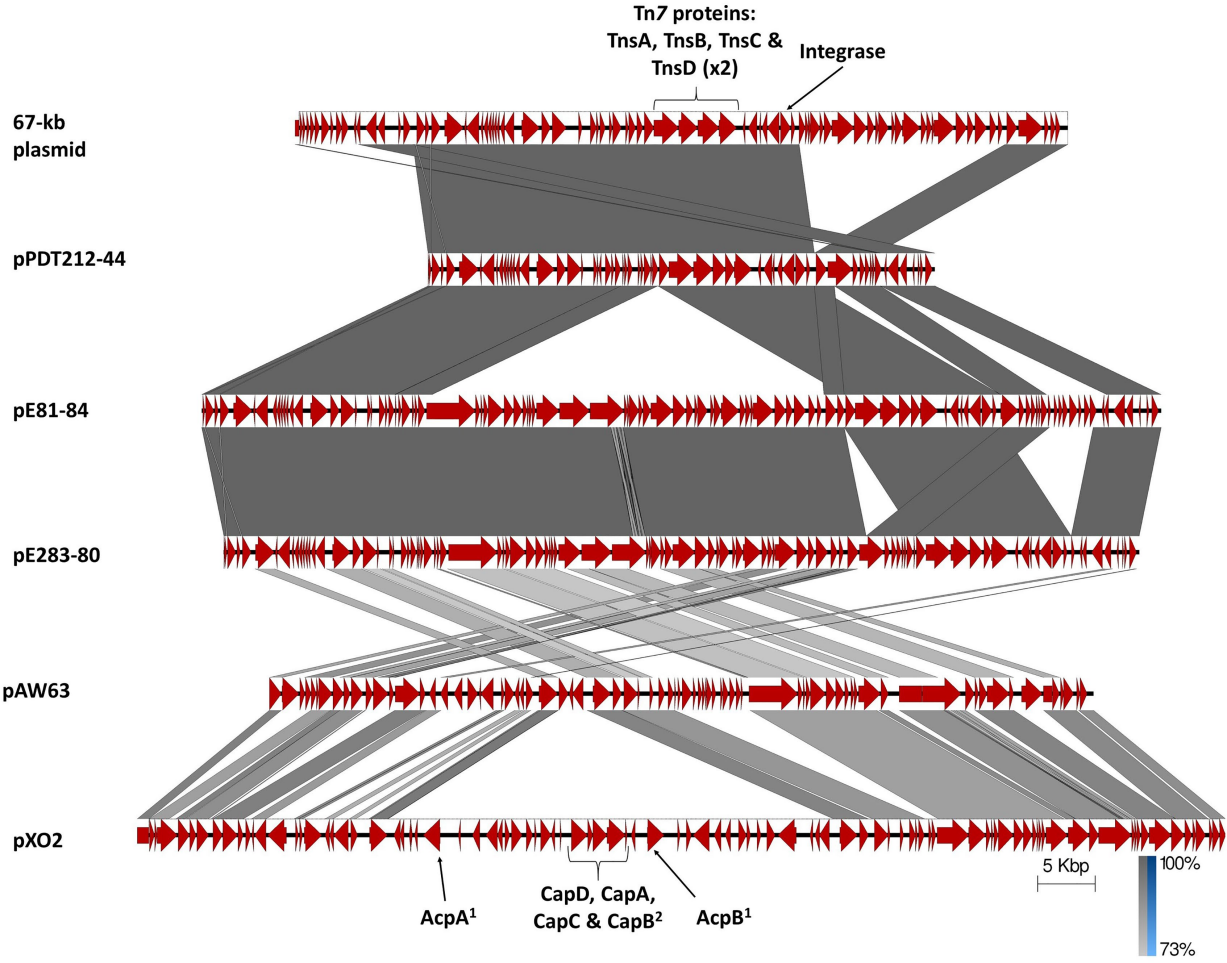
Linear alignment of the 67-kb plasmid (Stevens et al., 2019), pPDT212-44, pE81-84, pE283-80 from *B. cytotoxicus*, pAW63 (DQ025752.1) from *B. thuringiensis* sv. *kurstaki* HD73, and pXO2 (NC_007323.3) from *B. anthracis* (Wilcks et al., 1999; Van der Auwera et al., 2005). CDSs are represented by block arrows. Relevant functions are annotated above or below the corresponding gene. Darkening gray shading reflects increasing nucleotide BLAST sequence identity. Scale and identity percentage are indicated in the lower right-hand corner. Numbers 1 and 2 at the bottom refer to the *B. anthracis* capsule production regulators and components, respectively.

Three of the six large plasmids found in the analyzed *B. cytotoxicus* strains are related to each other. As shown in Figure 4, the 83,570-bp plasmid pE81-84 of strain E8.1 is closely related to the slightly smaller pE283-80 (79,734bp) from strain E28.3. They differ by a 3,836bp segment missing in the latter and by a different location of another segment. About half of these two elements is missing in pPDT212-44 (44,141bp), the only plasmid of the more distantly related strain PDT2.12 (Figures 2, 4). Interestingly, at the border of the missing region lies a Tn*7*-like element, which is present in all three plasmids. This putative *ca*. 7-kb transposon is reminiscent of a similar element found in strain ATCC 10987 of *B. cereus*, which was suggested to participate in the mobility of neighboring Genomic Islands (GI; Zhang and Zhang, 2008).

This plasmid trio is also distantly related to the conjugative plasmids pAW63 from *B. thuringiensis* sv. *kurstaki* HD73 and pBT9727 from *B. thuringiensis* sv. *konkukian*, and to the conjugation-deficient pXO2 from *B. anthracis* (Wilcks et al., 1999; Van der Auwera et al., 2005, 2008; Figure 4). In addition, they are partially related to the *B. cytotoxicus* p67 plasmid (Figure 4) reported by Stevens et al. (2019). Yet, whereas pE81-84 and pE283-80 contain a Type IV Secretion System (T4SS) region potentially involved in conjugative transfer (Van der Auwera and Mahillon, 2008), both pPDT212-44 and p67 are lacking this region.

**Figure 5:**
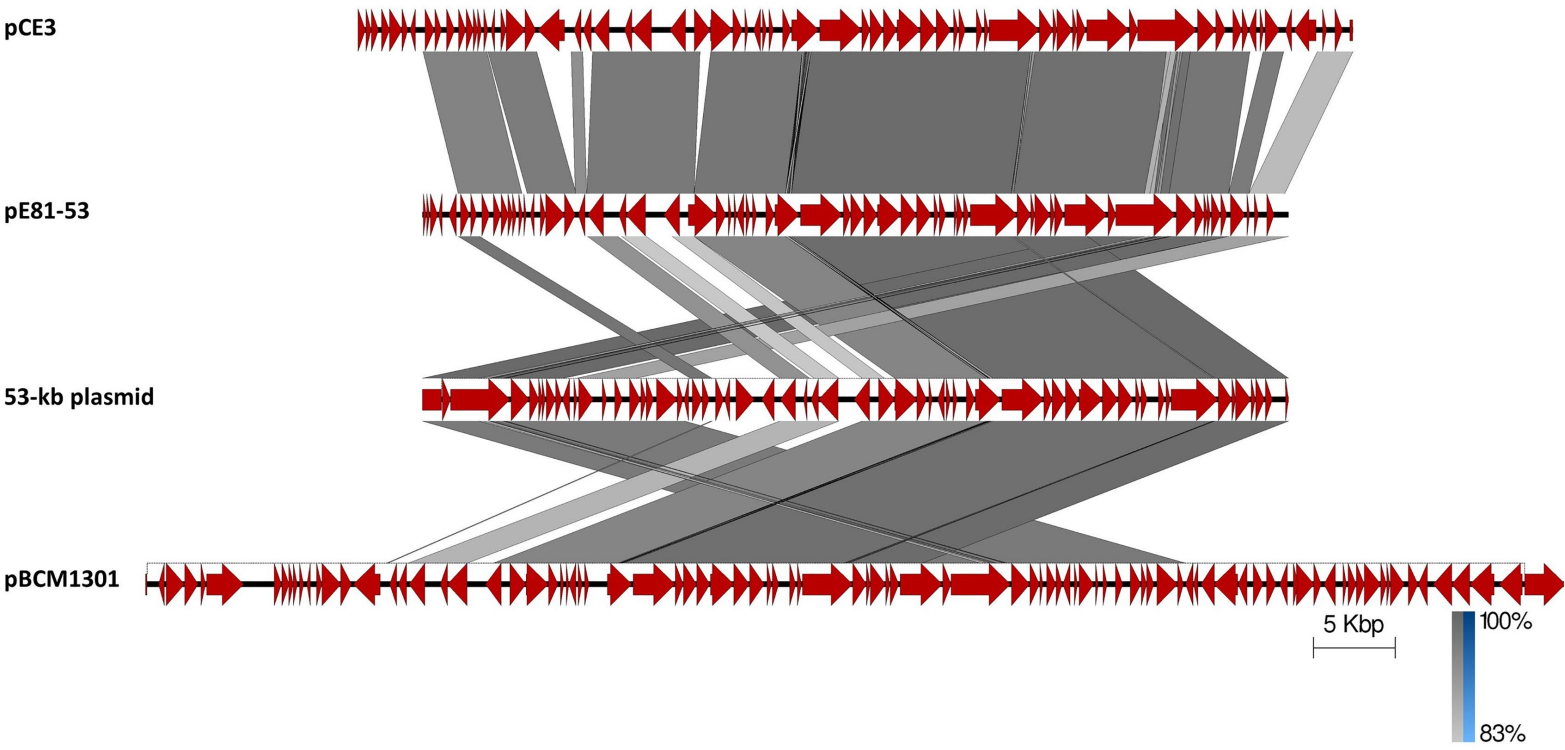
Linear alignment of pCE3 from *B. paranthracis* strain BC307 (CP047088.1), pE81-53 and the 53-kb plasmid (Stevens et al., 2019) from *B. cytotoxicus*, and pBCM1301 from *B. cereus* strain M13 (CP016361.1). CDSs are represented by block arrows. Darkening gray shading reflects increasing nucleotide BLAST sequence identity. Scale and identity percentage are indicated in the lower right-hand corner.

Strain E8.1 not only contains the potentially conjugative plasmid pE81-84, but also the 53,121bp plasmid pE81-53, which displays a *ca*. 20-kb “conjugation-related” region. This segment contains several genes coding for putative conjugal transfer proteins, including a TcpE-like protein found in the conjugative plasmid pCW3 from *Clostridium perfringens* (Wisniewski et al., 2015). It is related to the 53-kb plasmid found in clades B and C of *B. cytotoxicus* strains (Stevens et al., 2019), as well as to plasmids pCE3 from *Bacillus paranthracis* strain BC307 (NZ_CP047088.1) and pBCM1301 from *B. cereus* strain M13 (NZ_CP016361.1; Figure 5). In order to assess the potential mobility of pE81-53, filter-mating conjugation experiments (Hinnekens et al., 2019) were carried out between a streptomycin-resistant mutant strain E8.1 (donor) and a rifampicin-resistant mutant of E17.4 (recipient). After mating, the presence of pE81-53 in the recipient strain was detected *via* PCR. The results indicated that pE81-53 could be transferred at a frequency of about 10^−2^ transconjugants per donor (T/D), which suggests that it is a *bona fide* conjugative element.

The last two large plasmids reside in strain SM1.1: pSM11-43 (43,118bp) and pSM11-51 (51,478bp). With a query covering of 41% and an identity of 86%, pSM11-51 is related to plasmid pBc53 (NC_011971.1) from *B. cereus* strain Q1 (Xiong et al., 2009). As indicated below, pSM11-43 and pSM11-51 contain a significant proportion of their CDSs displaying homologies with those of Gram-positive prophages. Whether these elements are genuine plasmidial prophages or if they correspond to the insertion of prophages into resident plasmids remain to be tested.

### Other Mobile Genetic Elements

#### Prophages

PHAge Search Tool Enhanced Release was used to annotate phage-related proteins and to compare them to those available in the databases. A focus was granted to the six putative “intact” prophages detected in the strains. An average of 23 proteins related to the WBeta prophage, a siphovirus originally isolated from a *B. anthracis* strain (Schuch and Fischetti, 2006), were found on the chromosome of seven of the nine *B. cytotoxicus* strains. Potential Phi4B1-like elements (siphovirus from *B. thuringiensis* 4B1, NC028886) were also identified in eight of the nine strains, with the exception of E17.4. The CH_2 and E8.1 strains carry two copies of Phi4B1-like prophages, one “intact” with 22 and 23 related proteins and the second “partial” with only 13 Phi4B1-like proteins. Similarly, the number of identified Phi4B1-related proteins varied from 13 to 15 in E28.3, SM1.1, and SM2.8, respectively, suggesting the presence of “partial” Phi4B1-like element. Other potential prophages found on chromosomes are a Jenst-like prophage (siphovirus from *Brevibacillus laterosporus*; Merrill et al., 2015) only on SM1.1 chromosome, a GBSV1-like element (myovirus from *Geobacillus* sp.; Liu et al., 2009) only on CH_1 chromosome, and a B025-like prophage (siphovirus from *Listeria monocytogenes*; Dorsch et al., 2009) only on SM2.8 chromosome.

As for plasmidial prophages, as indicated above, pE283-14 is a putative 14-kb linear plasmidial prophage, similar to the tectiviral phages GIL01 and GIL16c isolated from *B. thuringiensis* (Verheust et al., 2005; Gillis and Mahillon, 2014a,b). A second potential plasmidial prophage is pSM11-43, the 43-kb extra-chromosomal element of strain SM1.1. It is related to the *L. monocytogenes* siphovirus B025 (Dorsch et al., 2009). Interestingly, as reported above, this element is integrated in the chromosome of strain SM2.8. This is potentially due to its dual capacity to integrate the chromosome using a site-specific integration mechanism or to replicate as a circular plasmidial prophage. In fact, this prophage carries an integrase coding gene, as well as recombination sites. For the chromosomal B025-like, it is inserted within a helicase-coding gene on the chromosome. The prophage-like region is flanked by *attL* and *attR* sites, located on the left and right ends of the prophage, whereas pSM11-43 holds an *attP*-like site.

Finally, it is worth mentioning that in strain SM1.1, the Jenst-like potential prophage is located both on its chromosome and on pSM11-51, where it constitutes *ca*. 55% of this 51-kb plasmid (Table 2). Within the chromosome, the Jenst-like prophage is inserted between a cation acetate symporter and a sodium/proline symporter PutP. Interestingly, Phi4J1, another siphovirus prophage from *B. thuringiensis* (NC029008), displayed similarities with the remainder region of pSM11-51, suggesting that this extrachromosomal element might also be a plasmidial prophage with distant relationship with both Jenst and Phi4J1 prophages. This plasmid also bares resemblance to the *Bacillus* phage BtS_BMBtp3 (NC028748) first described in *B. thuringiensis*.

#### Insertion Sequences, *B. cereus* Repeats, and Group II Introns

*Bacillus cytotoxicus* is a member of *B. cereus s.l.*, a group that has been shown to be rich in mobile genetic elements (Fayad et al., 2019). In this study, the sequenced isolates were mined for their IS elements, *bcr*, and group II introns. Using the ISsaga tool and a modified MegaBLASTn, IS and *bcr* elements and group II introns from newly sequenced isolates were compared to those of *B. cytotoxicus* strains NVH 391-98, CH_1, and CH_2 (genomic clades A, B, and C, respectively).

Elements belonging to nine IS families were found on the chromosomes, with no plasmidial IS elements recovered in the analyzed *B. cytotoxicus* genomes. As shown in Table 3, variations in the presence and copy number of IS families were observed. Out of the nine analyzed strains, (i) only SM1.1 and SM2.8 carry a copy of an IS*256*-like element and do not have an IS*4* family element, (ii) PDT2.12 lacks IS*30*- and IS*1182*-like elements, (iii) CH_1 does not carry an IS*110* element; and (iv) NVH 391-98 displays a much lower copy number (seven) of IS*3*-like elements compared to the other strains (from 17 to 24). As for the percentage of the genome covered by IS elements, it ranged between 0.79% for NVH 391-98 and 1.62% for SM1.1.

**Table 3:** Heat map of the distribution of IS families in the *B. cytotoxicus* genomes analyzed in this study.

*Bacillus cytotoxicus* strains (Clade)	IS*30*	IS*256*	IS*1182*	IS*200*/IS*605*	IS*4*	IS*6*	IS*3*	IS*21*	IS*110*	IS % of the chromosome
NVH 391–98* (A)	1	0	1	5	2	1	7	0	2	0.79
CH_1 (B)	2	0	3	5	2	0	17	0	0	1.19
CH_2 (C)	2	0	1	3	4	0	18	4	2	1.36
E8.1 (C)	2	0	1	3	4	0	19	4	2	1.44
E28.3 (C)	2	0	3	3	4	0	19	1	2	1.38
E17.4	2	0	3	5	1	1	22	2	2	1.60
PDT2.12	0	0	0	6	2	1	20	0	1	1.20
SM1.1 (D)	2	1	6	5	0	1	24	0	1	1.62
SM2.8 (D)	2	1	4	5	0	1	23	0	1	1.48

* Reference type-strain, previously analyzed in Fayad et al. (2019).

Proteins related to the Tn*7* transposon were found on the three related plasmids pPDT212-44, pE283-80, and pE81-84. The Tn7-encoded transposition genes *tnsA*, *B*, *C*, and *D* (two copies) as well as an integrase-coding gene are located on these plasmids (Figure 4). TnsA and B are at the heart of the transposition machinery since they mediate DNA strand breakage and joining, whereas TnsC and TnsD are regulators of transposition, also implicated in recognizing specific integration sites *attTn7* (Craig, 1996). As for the integrase, while its presence is not required for Tn*7* transposition, it plays a key role in acquiring gene cassettes as passenger genes associated with this transposon.

Concerning the *bcr*, 12 of the 18 known elements were found in the analyzed *B. cytotoxicus* genomes. Their genomic distribution is very similar among the strains, with small differences being noted for *bcr2* and *bcr5* (Supplementary Figure S1). *Bcr1* presented the highest copy number, from 57 in strain NVH 391-98 to 63 in strains SM1.1 and SM2.8. Finally, only one complete copy of *B.c*.I8, a group II intron encoding a 543 amino acid IEP originally found in *B. cytotoxicus* strain NVH 391-98, was found in the genomes of all six isolates.

## Discussion

Although it has been demonstrated that *B. cytotoxicus* strains constituted a remote cluster from the other *B. cereus* group members (Fagerlund et al., 2007), recent studies have shown that isolates of this species displayed noticeable chromosomal and plasmidial diversities (Koné et al., 2019; Stevens et al., 2019). The first *B. cytotoxicus* strain was isolated during a severe foodborne outbreak in France that led to three fatalities. Nevertheless, present data on this species suggest a strain-dependent, variable cytotoxicity. Indeed, a recent study suggested that the cytotoxic and fatal potential of this species might be lower than initially thought (Burtscher et al., 2021).

The current study extends insight into this intra-species genetic and genomic diversity. As for the other *B. cytotoxicus* genomes publicly available (Stevens et al., 2019), the six new strains sequenced in this study have chromosome sizes ranging from 4.1 to 4.2Mb. Also, their genomes contained up to 3.3% of plasmid DNA, with sizes ranging from 3.4 to almost 84kb. Compared to the four recently described clades (i.e., clades A–D, Stevens et al., 2019), two of our strains, E8.1 and E28.3, belong to clade C (Figure 2), that contained the majority of publicly available *B. cytotoxicus* genomes, while SM1.1 and SM2.8 pertain to clade D, together with strain AFSSA_08CEB44Bac (BioProject: PRJEB14962) isolated in France. The two remaining strains (i.e., PDT2.12 and E17.4) do not belong to previously described clades. This is in line with our recent study showing that these *B. cytotoxicus* isolates were the sole member of their RAPD patterns, while SM1.1 and SM2.8 displayed the same RAPD pattern (Koné et al., 2019), but differ by 13 chromosomal indels. Of note is the origin of these SM1.1 and SM2.8 strains that were isolated from two Moroccan soups: Harira and Chorba, respectively (Koné et al., 2019). However, it is worth mentioning that these instant soups contained potato-derived products as additives, which might be related to the presence of these bacteria.

Concerning the link between diversity and potential pathogenicity, it has been suggested that strains from clade A (which contains the original and reference type-strain NVH 391-98 and the highly cytotoxic CH_213) and from clade B were more likely to be cytotoxic compared to those of clades C and D (Stevens and Johler, 2020). However, the exact factor(s) and mechanism(s) responsible for the higher cytotoxicity displayed by these *B. cytotoxicus* strains remain(s) to be further explored.

The functional comparison showed that four out of six sequenced isolates (SM1.1, SM2.8, PDT2.12, and E17.4) possess enzymes implicated in the degradation of inositol, a polyalcohol mainly present in soil and plants. This catabolic operon has been found in environmental dwelling bacteria such as *Bacillus subtilis* or *Klebsiella aerogenes* (Yoshida et al., 2008).

As for other *B. cereus* group members, the *B. cytotoxicus* reference strain is not able to use galactose (Guinebretière et al., 2013), and analyses of publicly available sequences did not find any galactose degradation enzymatic pathway in their genomes. Surprisingly PDT2.12 contains sequences coding for galactose uptake and degradation enzymes. This hints that this isolate may have thrived in dairy environments and acquired these sequences from other galactose-using bacteria through horizontal gene transfer.

To avoid the taxonomic ambiguity of the *B. cereus* group, *B. cytotoxicus* genomes were searched for the presence of virulence genes typically used as markers for the entomopathogenic *B. thuringiensis* strains, i.e., those encoding crystal proteins or those toxic for insect larvae: the δ-endotoxins *cry*, cytolytic *cyt*, and the vegetative insecticidal protein coding genes *vip*. No entomopathogenic genes were found on the chromosomes or plasmids of the *B. cytotoxicus* strains.

More than a decade ago, the interest in phages preying on the *B. cereus* group was revived, bringing forth the questions about their diversity and potential implication in the ecology and adaptability of members of this group (Gillis and Mahillon, 2014a). While some have a lytic life cycle, others are lysogenic and can remain in a dormant state as prophages integrated into the chromosome, into plasmids or as circular/linear elements *aka* plasmidial prophages (Gillis and Mahillon, 2014a; Piligrimova et al., 2021). Prophages found in *B. cereus s.s*., *B. anthracis*, and *B. thuringiensis* strains have been extensively studied, thanks to the abundance of genomic sequences of these three species, in contrast to *B. cytotoxicus* genomes, still relatively new to the genomic field. In this study, the genomes of nine *B. cytotoxicus* strains were analyzed for the presence of prophages, *via* the online tool PHASTER. Six putative prophages were found to be “intact” on the chromosomes or plasmids. Interestingly, some prophages marked diverse regions on the chromosomes (regions 5, 6, 8, 10, and 11, Figure 1), making them a valuable addition to the bacteria’s genetic pool. For chromosomal and plasmidial prophages, the question of their activity and potential for a lytic cycle is still unanswered and requires further investigation. Nonetheless, for the plasmidial prophages, an added value could be their potential for horizontal genetic transfer, hence their status as “mobilizable.”

A special case is that of the prophage dubbed as B025-like, found as a plasmidial prophage in SM1.1, pSM11-43, and integrated in the chromosome of strain SM2.8. B025 originally isolated from a *Listeria* strain has the tools required for integration (*att* sequences and an integrase coding gene) and circularization (cohesive single-strand complementary *cos* ends). The capacity of a bacteriophage to exist in both integrated and excised forms was previously reported for a *Streptococcus pyogenes* M1 serotype phage SpyCIM1. The latter forms a chromosomal island integrated at a specific site of the chromosome, while the bacterium is in a stationary growth phase. However, once in exponential growth phase, SpyCIM1 excises from the chromosome and replicates as an independent plasmidial prophage (Nguyen and McShan, 2014; Utter et al., 2014). Another example is the circular plasmidial prophage pLUSID3, recently described in *B. thuringiensis* strain HER1410, which can also occur as fully integrated within the chromosome causing the disruption of a flagellar key component (Lechuga et al., 2020). Whether these B025-like elements are active or defective prophages and whether some are merely integrated into resident plasmids or are true plasmidial prophages will required further experiments.

Other mobile genetic elements were mined in the *B. cytotoxicus* genomes, including IS elements. Although no plasmidial IS were recovered, the variability of chromosomal IS elements between the different strains and clades was evident, with NVH 391-98 presenting the lowest number of IS. Surprisingly, 1.62% of the chromosome of SM1.1 is covered by IS, the highest number for the nine strains, which is higher than the average 1.1% of *B. thuringiensis*, keeping in mind that for the latter species, IS prevalence and diversity are dominantly plasmidial (Fayad et al., 2019).

The plasmidome showed the greatest diversity among the six sequenced *B. cytotoxicus* strains, including four small plasmids and six large ones, whose prevalence and distribution are summarized in Table 4. While some plasmids were unique to a particular strain, e.g., the 3- and 4-kb plasmids of E28.3, others were more or less similar to plasmids carried by other strains. For the 12-kb plasmids, one set (12a) is identical in SM1.1 and SM2.8, while the other (12b) is identical between SM1.1 and SM2.8 but is closely related to pE174-12 and the 7-kb pBC9801 element of the reference strain NVH 391-98. The main difference between the 7-kb, 12b, and pE174-12 plasmids is the replication protein (Figure 3). However, one of the main shared regions is encoding a Fibronectin type III domain-containing protein. This domain mediates protein–protein interaction and is potentially involved in the correct positioning of a protein’s active domain (Campbell and Spitzfaden, 1994). Although its exact role remains unclear, it is often associated with enzymes involved in the degradation of recalcitrant polysaccharides such as chitin, as shown for the chitinase of *B. thuringiensis* (Juárez-Hernández et al., 2019).

**Table 4:** Distribution of the various plasmids across the different *B. cytotoxicus* clades and strains.

Clade	Strains	pE283-3 (3,421)	pE283-4 (3,662)	pBC9801 (7,135)	pSM11-12b (11,581)	pSM11-12a (11,640)	pE174-12 (11,673)	pE283-14 (14,402)	pSM11-43 (43,118)	pPDT212-44 (44,141)	pE81-53 (53,121)	p53 (53kb)	p67 (67kb)	pSM11-51 (51,478)	pE283-80 (79,734)	pE81-84 (83,570)
A	NHV 391-98															
	CH_13															
B	CH_1															
	CH_23															
C	CH_2															
	E8.1															
	E28.3															
D	SM1.1															
	SM2.8															
-	E17.4															
-	PDT2.12															

The colors indicate that the corresponding plasmids are (partially) similar; the details of their relationships are shown in Figures 3–5. Also, note that pE283-14 is a putative plasmidial prophage. The NVH and CH strains, as well as the p53 and p67 plasmids, have been described in Stevens et al. (2019).

The most prevalent plasmid, or plasmidial region, is pPDT212-44, to which similarities are found on five other plasmids, in five strains from two clades, and one not placed in a particular clade (Table 4; Figure 4). Finally, the 53-kb conjugative plasmid pE81-53 shows similarities with the other 53-kb elements in previously reported *B. cytotoxicus* strains, implying their conjugative potential as well (Figure 5; Stevens et al., 2019). In total, the 53-kb element was found in four strains from two different clades, B and C (Table 4).

Another quite interesting feature of the small plasmids is the presence on pSM11-12a of strain SM1.1 (as well as on strain SM2.8) of three genes highly similar to the *gakABC* locus found in *Lactococcus garvieae* that code for the three-peptide bacteriocin Garvicin KS (Ovchinnikov et al., 2016). This bacteriocin and homologues found in *B. cereus* strains are active against several other Gram-positive bacteria including members of the *Bacillus*, *Enterococcus*, *Listeria*, and *Streptococcus* genera. Intriguingly, the homology extends outside this locus and includes neighboring genes such as putative ABC transporter and recombinase/integrase-like genes, suggesting they are part of a mobile genetic element. The activity of this Garvicin KS-like bacteriocin, also referred to as Cereucin in the case of the *B. cereus* strains (Ovchinnikov et al., 2016), on other bacteria is currently under investigation.

The aim of this study was to extend the understanding about the intra-species diversity of *B. cytotoxicus* through WGS and comparative genomic analyses of six isolates. The SNP-based phylo-dendrogram, as well as the ANI, showed that two isolates from instant soup fitted in the clade D, two from potato flakes were classified in clade C, and the last two, also from potato flakes, formed a separated cluster from the other clades. The plasmidial diversity is also in line with previous studies. The presence of sequences coding for inositol degradation in four isolates genomes and galactose uptake and degradation enzymes hinted the ecological niche of *B. cytotoxicus*. Nevertheless, in order to extend the insight on knowledge about the genetic diversity of *B. cytotoxicus* and its ecological niche, there is a need to sequence and study more isolates from matrices other than potato products.

## Data Availability Statement

The datasets presented in this study can be found in online repositories. The names of the repository/repositories and accession number(s) can be found at: https://www.ncbi.nlm.nih.gov/, PRJNA684687.

## Author Contributions

KK, NF, AG, and JM contributed to conceptualization, validation, methodology, and writing – review and editing. KK, AG, and NF provided software and were involved in investigation. KK, NF, and JM contributed to formal analysis, data curation, writing – original draft preparation, and visualization. JM was involved in resources, supervision, project administration, and funding. All authors contributed to the article and approved the submitted version.

## Funding

This work was supported by the International Office for Cooperation of the *Université catholique de Louvain* (UCLouvain; Bursaries to KK and NF), the Research Department of the Communauté française de Belgique (Concerted Research Action, ARC 17/22-084), and the National Fund for Scientific Research (FNRS, Belgium; research grant FNRS-CDR J.0144.20 to JM and research position FNRS 1.B208.16 to AG).

## Conflict of Interest

The authors declare that the research was conducted in the absence of any commercial or financial relationships that could be construed as a potential conflict of interest.

## Publisher’s Note

All claims expressed in this article are solely those of the authors and do not necessarily represent those of their affiliated organizations, or those of the publisher, the editors and the reviewers. Any product that may be evaluated in this article, or claim that may be made by its manufacturer, is not guaranteed or endorsed by the publisher.

## Footnotes

^1^ https://www.ncbi.nlm.nih.gov/genome/genomes/2491?

^2^ http://bio-bwa.sourceforge.net

^3^ https://github.com/rrwick/Porechop

^4^ https://sourceforge.net/projects/bbmap/
