# Supplementary material for: Bacillus cytotoxicus Genomics: Chromosomal Diversity and Plasmidome Versatility
Source: Front Microbiol. 2021 Dec 9;12:789929. doi: 10.3389/fmicb.2021.789929 (PMC8725734; doi:10.3389/fmicb.2021.789929)
Supplement: Supplementary file 1 [file Table_1.docx]

Supplementary Material

**Table S1:** Relevant features of the *B. cytotoxicus* plasmids described in this study.

| **Plasmid** | **Plasmid relevant features (putative encoded proteins)** |
| --- | --- |
| **pE81-84**  (83,570 bp) | DNA (cytosine-5-)-methyltransferase (*Hha*I) and DNA cytosine methyltransferase (*Hpa*II)  Site-specific DNA-methyltransferase (YhdJ)  Peptide ABC transporter substrate-binding protein  Response regulator aspartate phosphatase (RapF/H) and HTH transcriptional regulator (PagR-like)  Sporulation initiation inhibitor Soj  Replication initiation, primase C-ter domain-containing  pXO2-28/29/30-like  Flp pilus assembly complex ATPase component TadA (Type II/IV secretion system)  TrbC/VirB2 family protein  T4SS conjugative DNA transfer  S-layer family protein  Murein DD-endopeptidase MepH prec. - Lysozyme-like  ImmA/IrrE family metallo-endopeptidase  Metal-dependent hydrolase (LexA-binding, inner membrane-associated putative hydrolase)  DNA primase dnaG (?)  Type III toxin-antitoxin ToxN/AbiQ family toxin  Type IA DNA topoisomerase  Tn*7* transposition endonuclease TnsA, TnsB (DDE-type), TnsC and 2 TnsD (2)  CDGSH iron-sulfur domain-containing protein  SDR family oxidoreductase  Cyclopentanol dehydrogenase cpnA_2  Universal stress protein  YeiH family putative sulfate export transporter and LysR family transcriptional regulator  Iron-sulfur cluster biosynthesis family protein  Type IA DNA topoisomerase  CPBP family intra-membrane metallo-protease, Abi, CAAX protease self-immunity; pfam02517  Iron ABC transporter permease  Tyrosine recombinase XerC, Site-specific integrase  Tn*552* DNA-invertase Bin3, recombinase protein  HTH DNA binding domain of transcription regulators, MerR superfamily |
| **pE81-53**  (53,121 bp) | Sporulation-specific extracellular. nuclease precursor  DNA-invertase resolvase  Molecular chaperone DnaJ - Nuclease, RecB  DNA segregation ATPase FtsK/SpoIIIE  Replication-relaxation family protein - Mob protein  Site-specific integrase, Y-recombinase  ParM/StbA plasmid segregation protein  Replicase RepFR55  HAMP domain-containing histidine kinase (TC sensor)  Response regulator transcription factor  HTH transcriptional regulator  HTH transcriptional regulator, XRE family like (2)  S-layer homology domain-containing protein  Conjugal transfer protein  Flp pilus assembly protein CpaB Pilus assembly protein, ATPase of CpaF family  T4SS conjugative DNA transfer family protein  ATP-binding protein (ATPase)  Replication-relaxation (Mob) family  Conjugal transfer protein  Conjugal transfer protein (TcpE-like)  ATP-binding protein (recombinase ?)  Mannosyl-glycoprotein endo-beta-N-acetyl-glucosaminidase, Murein DD-endo-peptidase MepM  DNA repair protein RadC |
| **pE174-12**  (11,673 bp) | ATP-binding protein (ATPase)  Fibronectin type III domain-containing protein  Replication protein - pGI3 family  Fibronectin type III domain-containing protein |
| **pE283-80**  (79,734 bp) | Site-specific DNA Recombinase family protein  Phage integrase  HTH domain-containing protein  Iron-sulfur cluster biosynthesis ?  LysR family transcriptional regulator  YeiH family putative sulfate export transporter  Universal stress protein  MFS transporter  SDR family oxidoreductase  CDGSH iron-sulfur domain-containing  Tn*7* transposition endonuclease TnsA, TnsB (DDE-type), TnsC and 2 TnsD (2)  Iron ABC transporter permease  CPBP family intramembrane metalloprotease  Type IA topoisomerase  Type III toxin-antitoxin system ToxN/AbiQ toxin  DUF3991 domain-containing protein - LtrC-like  Membrane-bound metal-dependent hydrolase YdjM, induced during SOS response  ImmA/IrrE metallo-endopeptidase, pXO2-80-like  ImmA/IrrE metallo-endopeptidase, LtrC-like protein  Lysozyme family protein (pAW63_6/8-like)  Putative ATPase TraE  pXO2-10-like, pXO2-11-like, pXO2-12-like  pXO2-14-like  T4SS conjugative DNA, TraG/Tra-like, pXO2-16-like  TrbC/VirB2 protein  Type II/IV secretion system ATPase subunit, Flp pilus assembly complex ATPase component TadA  pXO2-27-like  Fusion protein pXO2-28/29/30-like  DNA topoisomerase III (?)  Prophage HTH protein (?)  pXO2-37-like  Plasmid replication protein, pXO2-38-like  Plasmid partitioning/segregation protein ParA  Transcriptional regulator, ArsR family  Response regulator aspartate phosphatase RapH  General stress protein  ABC transporter, substrate-binding protein  Site-specific DNA-methyltransferase  DNA cytosine methyltransferase, HgiDII  DNA (cytosine-5-)-methyltransferase |
| **pE283-14**  (14,402 bp) | Putative plasmidial tectivirus |
| **pE283-4**  (3,662 bp) | Rep - *B. thuringiensis* sv. *israelensis* pTX14-1-like  Possible lipoprotein |
| **pE283-3**  (3,421 bp) | Mob related to the streptococcal plasmid pMV158 Mob  Rep protein RepL - Related to plasmid pE194 and pSN2 Rep |
| **pPDT212-44**  (44,141) | Primase C-terminal domain-containing protein - Rep63A-like  pXO2-28/29/30-like protein  Tn*7* transposition endonuclease TnsA, TnsB (DDE-type), TnsC and 2 TnsD (2)  CDGSH iron-sulfur domain-containing protein  SDR family oxidoreductase  Cyclopentanol dehydrogenase cpnA_2  Universal stress protein  YeiH family putative sulfate export transporter and LysR family transcriptional regulator  LysR family transcriptional regulator  Iron-sulfur cluster biosynthesis family protein  Type IA DNA topoisomerase - DNA topoisomerase III  CPBP family intra-membrane metallo-protease  Tyrosine recombinase XerC  Tn*552* DNA-invertase Bin3 - recombinase  DNA (cytosine-5-)-methyltransferase  DNA cytosine methyltransferase  DNA adenine methyltransferase YhdJ  Dipeptide-binding protein DppE precursor  Response regulator aspartate phosphatase F - RapF  Transcriptional repressor PagR  Sporulation initiation inhibitor protein Soj |
| **pSM11-51**  (51,478 bp) | 73 CDS (54 hypothetical)  Putative plasmidial prophage related to the *Brevibacillus* Jenst and *B. thuringiensis* Phi4J1 |
| **pSM11-43**  (43,118 b) | 70 CDS (20 hypothetical)  Putative plasmidial prophage related to *Listeria* prophage BO25 |
| **pSM11-12a**  (11,640 bp) | Mob protein - pfam01076  Rep protein of an unknown family (related to Mar transcriptional regulator)  MerR transcriptional regulator  DUF1878 family protein  S8 family peptidase  AAA family ATPase  Y-recombinase/integrase  Three GatB family leaderless bacteriocins  ABC transporter |
| **pSM11-12b**  (11,581 bp) | Rep protein of an unknown family  Fibronectin type III domain protein  ATP-binding protein |
| **pSM28-12a**  (11,640 bp) | Identical to pSM11-12a |
| **pSM28-12b**  (11,581 bp) | Identical to pSM11-12b |
