# Supplementary material for: Bacillus cytotoxicus Genomics: Chromosomal Diversity and Plasmidome Versatility
Source: Front Microbiol. 2021 Dec 9;12:789929. doi: 10.3389/fmicb.2021.789929 (PMC8725734; doi:10.3389/fmicb.2021.789929)
Supplement: Supplementary file 2 [file Image_1.pdf]

Supplementary Material

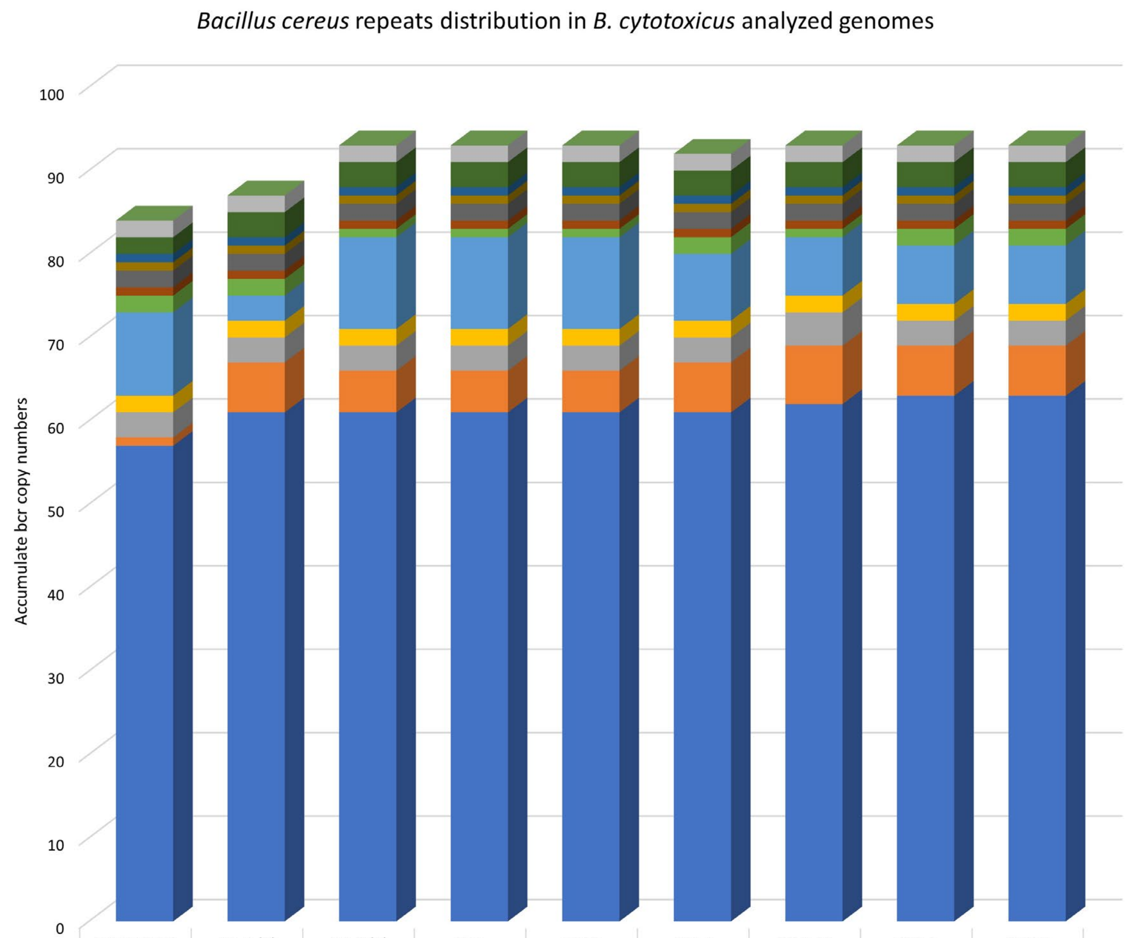

	NVH 391-98 (A)	CH_1 (B)	CH_2 (C)	E8.1	E28.3	E17.4	PDT2.12	SM1.1	SM2.8
bcr18	0	0	0	0	0	0	0	0	0
bcr17	0	0	0	0	0	0	0	0	0
bcr16	0	0	0	0	0	0	0	0	0
bcr15	2	2	2	2	2	2	2	2	2
bcr14	0	0	0	0	0	0	0	0	0
bcr13	0	0	0	0	0	0	0	0	0
bcr12	2	3	3	3	3	3	3	3	3
bcr11	1	1	1	1	1	1	1	1	1
bcr10	1	1	1	1	1	1	1	1	1
bcr9	2	2	2	2	2	2	2	2	2
bcr8	1	1	1	1	1	1	1	1	1
bcr7	0	0	0	0	0	0	0	0	0
bcr6	2	2	1	1	1	2	1	2	2
bcr5	10	3	11	11	11	8	7	7	7
bcr4	2	2	2	2	2	2	2	2	2
bcr3	3	3	3	3	3	3	4	3	3
bcr2	1	6	5	5	5	6	7	6	6
bcr1	57	61	61	61	61	61	62	63	63

Figure S1: Distribution of the total *BCR* copies in the complete genomes of seven analysed *B. cytotoxicus* strains. The copy number of each *BCR* element is shown in the bar graph and Table. Colour key is shown next to each *BCR* element in the Table.
